# Absence of *Neospora caninum* DNA in Human Clinical Samples, Spain

**DOI:** 10.3201/eid2506.181431

**Published:** 2019-06

**Authors:** Rafael Calero-Bernal, Pilar Horcajo, Marta Hernández, Luis Miguel Ortega-Mora, Isabel Fuentes

**Affiliations:** Complutense University of Madrid, Madrid, Spain (R. Calero-Bernal, P. Horcajo, L.M. Ortega-Mora);; Carlos III Institute of Health, Madrid (M. Hernández, I. Fuentes)

**Keywords:** *Neospora caninum*, toxoplasmosis, neosporosis, parasites, PCR, Spain

## Abstract

Low antibody titers to *Neospora caninum* have been reported in humans, but infection has not been confirmed. We used *N. caninum*–specific PCR to test 600 clinical samples from patients with toxoplasmosis signs but *Toxoplasma gondii*–negative PCR results. We did not detect *N. caninum* DNA, demonstrating it is an unlikely opportunistic zoonotic agent.

The coccidian parasite *Neospora caninum* (Apicomplexa: Sarcocystidae) is a major abortifacient agent in ruminants, especially cattle. It is phylogenetically close to *Toxoplasma gondii* (*1*), a parasite of high prevalence in humans, but biologically different. *N. caninum* parasites have a restricted host range but can infect primates (*2**,**3*).

*N. caninum* infection causes neuromuscular disease in dogs and reproductive disorders in ruminants, causing fetal loss due to vertical transfer of parasites during acute infections or reactivation of chronic infections. Clinical neosporosis in animals resembles the disease outcome of toxoplasmosis (*1*). 

*N. caninum* parasites have been successfully cultured in human cell lines, but low antibody titers of unconfirmed specificity against *N. caninum* have been reported in human serum samples (*1*,*4*,*5*). The significance of these findings is uncertain because neither parasite DNA nor viable parasites have been demonstrated in human tissues. Unconfirmed reports of *N. caninum*–specific antibodies in the human population (*4**,**5*) prompted us to test specifically for *Neospora* DNA in human clinical specimens and assess its possible role in human illness.

We obtained 600 DNA samples from a collection of anonymized human clinical samples from the National Registry of Biobanks (no. C.0004715) in Spain that were deemed exempt from a second ethics approval. Our criteria for selection included any pregnancy-related disorder affecting women or fetuses, toxoplasmosis-like clinical signs or suspicion of toxoplasmosis, and negative results for *T. gondii*–specific real-time PCR (*6*) and nested PCR (*7*) (Table).

**Table:** Types of samples analyzed and demographic and clinical data for 600 patients tested for *Neospora caninum* parasites, Spain*

Characteristics	No. (%)
Sample type	
Amniotic fluid	267 (44.5)
Cerebrospinal fluid	113 (18.8)
Blood	100 (16.7)
Placental tissue	51 (8.5)
Bronchoalveolar lavage	25 (4.2)
Urine	17 (2.8)
Brain biopsy	12 (2.0)
Aqueous humor	4 (0.7)
Fetal tissues and fluids	4 (0.7)
Lymph node aspirate	3 (0.5)
Vitreous humor	2 (0.3)
Bone marrow aspirate	1 (0.2)
Hepatic abscess aspirate	1 (0.2)

Patient demographic information	
Sex	
Unknown	6 (1.0)
M	135 (22.5)
F	459 (76.5)
Of childbearing age, 17–42 y	333 (55.5)
Age, y	
>1, average 36.1	481 (80.2)
<1	116 (19.3)

Native country	
Spain	382 (63.7)
Other†	36 (6.0)
Unknown	182 (30.3)
Patient clinical information	
Immune status	
Immunocompetent	458 (76.3)
Immunodepressed or immunosuppressed	83 (13.8)
HIV/AIDS	19 (22.9)
Chemotherapy	29 (34.9)
Organ transplant	35 (42.2)
Unknown	59 (9.8)
Pregnancy-related disorders	454 (75.7)
Seroconversion–infection suspicion and lymphadenopathy	418 (92.1)
Spontaneous abortion	27 (5.9)
Ophthalmic	4 (0.9)
Other fetal signs‡	5 (1.1)
Neurologic and ocular symptoms and conditions§	108 (18.0)
Neurologic condition with ocular signs	8 (7.4)
Ophthalmic only	3 (2.7)
General signs, n = 38	38 (6.3)
Pneumonia	24 (63.2)
Lymphadenopathy	2 (5.3)
Hematologic or oncologic	12 (31.6)

*Patients were from 12 regions: Andalusia, Aragon, Asturias, Balearic Islands, Cantabria, Castile-La Mancha, Castile and Leon, Extremadura, Galicia, Madrid, Navarre, Valencia.  †Africa, 8; Asia, 1; Europe, 8; Latin America, 19. ‡Anencephaly, malformations, and microcephaly. §Neurologic symptoms and conditions include ataxia, disorientation, sudden blindness, encephalitis, calcifications, and intracranial space occupying lesions; ophthalmic symptoms and conditions include chorioretinitis, panuveitis, posterior uveitis, and vitritis.

We isolated total DNA using a QIAamp DNA Mini Kit (QIAGEN, https://www.qiagen.com) and used a single-tube nested PCR to amplify the *N. caninum* internal transcribed spacer 1 region using external primers NN1–NN2 and internal primers NP1–NP2, as previously described (*8*,*9*). We expected a diagnostic 249-bp fragment. In each batch of amplifications, positive PCR controls included genomic DNA of 10, 1, and 0.1 *N. caninum* tachyzoites. Using these PCR methods, we found that the analytical sensitivity was <1 tachyzoite of *Neospora* spp. or *T. gondii*.

We did not detect *N. caninum*–specific DNA in the samples analyzed. Previously, transplacental neosporosis was experimentally demonstrated in rhesus macaques (*Macaca mulatta*) in the United States (*2*,*3*). A literature review summarized reports of unconfirmed presence of antibodies against *N. caninum* in patients with neurologic disorders, pregnant women, and healthy people, including blood donors (*1*). Findings of *N. caninum* IgG in HIV-infected patients from Brazil and France (*4*,*5*) are of special interest because of possible association with *T. gondii* infections.

We believe *N. caninum* parasites are an unlikely opportunistic zoonotic agent. Application of direct methods for parasite detection in a greater number of samples from HIV-positive patients should complement unclear serologic findings to fully dispel suspicion of human neosporosis.
